# The association of ınsomnia with fatigue and mood in postmenopausal osteoporosis

**DOI:** 10.1590/1806-9282.20250426

**Published:** 2025-09-19

**Authors:** Büşra Şirin Ahısha, Nurdan Paker, Nur Kesiktaş, Nazlı Derya Buğdaycı, Sedef Ersoy, Yiğit Can Ahısha

**Affiliations:** 1 Beylikdüzü State Hospital – Istanbul, Turkey.; 2 University of Health Sciences, Istanbul Physical Therapy and Rehabilitation Training and Research Hospital – Istanbul, Turkey.; 3 University of Health Sciences, Bakırköy Dr. Sadi Konuk Education and Research Hospital – Istanbul, Turkey.

**Keywords:** Osteoporosis, Postmenopause, Insomnia, Fatigue

## Abstract

**OBJECTIVE:**

The aim of the study was to investigate the prevalence of insomnia in postmenopausal osteoporotic women. It also evaluated the relationship between insomnia, fatigue, and mood disorders.

**METHODS:**

This cross-sectional study was conducted with 200 postmenopausal osteoporotic women aged 50–75 years. Insomnia severity was assessed using the Insomnia Severity Index, while fatigue and mood disorders were measured using the Fatigue Severity Scale and the Hospital Anxiety and Depression Scale, respectively. The participants were divided into two groups based on the Insomnia Severity Index scores, using a cutoff of 15. We then analyzed the clinical characteristics of these two groups, focusing on the relationship between insomnia severity and factors such as fatigue, anxiety, depression, and bone mineral density.

**RESULTS:**

Among the participants, 20.5% were clinically diagnosed with insomnia (Insomnia Severity Index≥15). Sleep initiation difficulties were reported by 73.5%, sleep maintenance difficulties by 57.5%, and early awakening by 45%. Insomnia severity was significantly correlated with higher levels of fatigue (r=0.342, p<0.001), depression (r=0.297, p<0.001), and anxiety (r=0.323, p<0.001). There was no significant correlation between insomnia and bone mineral density or T-scores.

**CONCLUSION:**

Insomnia is common in postmenopausal osteoporotic women and is closely linked to higher fatigue, anxiety, and depression levels. Early detection and management of insomnia in this population are essential to reduce fall risk and prevent fractures.

## INTRODUCTION

Insomnia is defined as a disorder affecting sleep initiation, maintenance, duration, or quality, impairing functionality despite adequate environmental and physical conditions^1^. Its prevalence is higher in women than men and increases with age, becoming more pronounced after menopause. Studies attribute insomnia in menopausal women to "hot flashes," especially when they disrupt sleep at night. Additionally, conditions such as sleep apnea, anxiety, depression, and declining health, which are more common with age and in the postmenopausal period, are also linked to insomnia^1,2^.

Metabolic syndrome, obesity, and endocrine disorders are various medical conditions that can lead to sleep problems due to their physical and psychological effects. Osteoporosis is also one of these conditions, and it is thought to affect sleep quality through its association with vertebral compression fractures, pain, depression, and anxiety symptoms. Insomnia and daytime sleepiness are known to increase the risk of falls and related osteoporotic fractures. Early identification and management of sleep disturbances may help reduce these risks^3^.

Various methods have been used to assess sleep problems in the postmenopausal period, including "yes/no" patient questionnaires, the Pittsburgh Sleep Quality Index^4^, and the Women's Health Initiative Insomnia Rating Scale (WHIIRS)^5^. This study used the Insomnia Severity Index (ISI) for several reasons. The ISI is a reliable and widely used tool that captures both the severity and impact of insomnia based on patients’ subjective experiences. It is also brief, easy to administer, and practical for clinical use.

Most of the existing studies in the literature focus on sleep problems during the postmenopausal period, while the number of studies specifically evaluating osteoporotic patients remains very limited^5,6^. To our knowledge, no prior study has used the ISI to assess insomnia in postmenopausal osteoporosis. This study aims to fill that gap by examining the frequency of sleep problems and their associations with fatigue, depression, and anxiety in this population.

## METHODS

### Study design

This cross-sectional study included 200 women aged 50–75 years with postmenopausal osteoporosis who attended the outpatient clinics of Beylikdüzü State Hospital and Istanbul Physical Therapy and Rehabilitation Training and Research Hospital between May 19, 2024, and June 19, 2024. Ethical approval was obtained from the Istanbul Physical Therapy and Rehabilitation Training and Research Hospital Ethics Committee (protocol number 2024-16). Exclusion criteria included fibromyalgia, chronic fatigue syndrome, chronic decompensated cardiac, renal, or hepatic failure, known psychiatric or rheumatologic diseases, other neurological disorders, and the use of medications known to affect physical performance, fatigue, mood, or sleep quality. These medications included hypnotics, selective serotonin reuptake inhibitors, serotonin-norepinephrine reuptake inhibitors, tricyclic antidepressants, and other centrally acting agents that may influence sleep regulation. In total, 38 individuals were excluded during screening: 12 for psychiatric disorders (e.g., major depression, anxiety), 9 for relevant medication use, 6 for rheumatologic diseases, 5 for neurological conditions, 3 for fibromyalgia, and 3 for decompensated organ failure.

At the start of the study, informed consent was obtained from all participants. Sociodemographic data (gender, height, weight, body mass index [BMI], and occupation), comorbidities, smoking, daily coffee intake, low-energy fracture history, and regular exercise habits were recorded. Regular exercise was defined as planned physical activity for at least 30 min, three times per week. Pain presence and severity were assessed, and recent calcium and 25-hydroxy vitamin D levels were documented. Sleep problems were assessed with the ISI, depression and anxiety symptoms with the Hospital Anxiety and Depression Scale (HADS)^7^, and fatigue levels with the Fatigue Severity Scale (FSS)^8^.

## OUTCOMES

### Insomnia Severity Index (ISI)

The ISI is a seven-item scale developed by Morin et al.^9^ to assess insomnia symptoms and their impact over the past month. Total scores range from 0 to 28: 0–7 indicates no insomnia, 8–14 sub-threshold insomnia, 15–21 moderate insomnia, and 22–28 severe insomnia. It evaluates sleep onset and maintenance difficulties, early awakening, satisfaction with sleep, and the effect of sleep problems on daily functioning and psychological well-being. A score of ≥15 indicates clinical insomnia.

### Fatigue Severity Scale (FSS)

The questionnaire consists of nine items evaluating the severity and impact of fatigue. Each item is rated on a scale from 0 (strongly disagree) to 7 (strongly agree). The total score is calculated as the average of all responses, ranging from 0 to 7, with higher scores indicating greater fatigue severity^8^.

### Visual Analog Scale (VAS)

To evaluate the extent of chronic pain among participants, we employed the VAS^10^. This scale is anchored with 0 indicating the absence of pain and 100 indicating the highest intensity of pain.

### Hospital Anxiety and Depression Scale (HADS)

This 14-item scale assesses anxiety and depression, with seven items each rated from 0 to 3 (maximum 21 per subscale). Scores ≥8 indicate significant depression, and scores ≥11 indicate significant anxiety^7^.

### Statistical analysis and sample size

Sleep disturbances in osteoporotic women have been reported at rates up to 84.6%^11^. Based on this, a sample size calculation using G*Power 3.1.9.4 indicated that at least 200 patients were needed. Normality was assessed with Shapiro-Wilk and Kolmogorov-Smirnov tests. Quantitative data were presented as mean±SD, and qualitative data as frequencies and percentages. Non-normally distributed data were analyzed with the Mann-Whitney U test and correlations with Spearman's test. Logistic regression identified factors independently associated with insomnia, and mediation analysis explored indirect effects. A p<0.05 was considered significant. Analyses were performed using IBM Statistical Package for the Social Sciences (SPSS) 21.

## RESULTS

The mean age was 62.13±7.10 years, and BMI was 26.74±4.57. Osteoporosis duration averaged 5.77±6.24 years. Bone mineral density (BMD) values, T-scores, and serum vitamin D and calcium levels are summarized in Table 1. Mean HADS scores were 6.96±4.59 (depression) and 7.85±4.59 (anxiety), and the mean ISI score was 8.25±6.78. Sleep complaints included difficulty falling asleep (73.5%), maintaining sleep (57.5%), and early awakening (45%) (Table 1).

**Table 1:** Clinical and sleep-related characteristics of participants.

		Mean±SD/n (%)	Median (minimum–maximum)
Age (years)		62.13±7.10	62 (47–75)
BMI (kg/m^2^)		26.74±4.57	26.08 (17.75–43.28)
Occupation	Employed	7 (3.5%)	
	Retired	74 (37.0%)	
	Housewife	119 (59.5%)	
Femoral neck BMD (g/cm^2^)		0.72±0.09	0.72 (0.45–0.99)
L1–L4 BMD (g/cm^2^)		0.74±0.08	0.74 (0.60–1.12)
L1–L4 T score		-2.88±0.54	-2.90 (-4.50 to -0.20)
Femoral neck T score		-1.63±0.78	-1.60 (-3.50 to 0.70)
25-OH-D (ng/mL)		29.77±13.29	28.95 (6–75)
Calcium		9.71±1.21	9.65 (8.20–25.40)
ISI		8.25±6.78	7 (0–28)
Difficulty initiating sleep	None	53 (26.5)	
	Mild	39 (19.5)	
	Moderate	59 (29.5)	
	Severe	30 (15.0)	
	Very severe	19 (9.5)	
Difficulty maintaining sleep	None	85 (42.5)	
	Mild	26 (13.0)	
	Moderate	45 (22.5)	
	Severe	35 (17.5)	
	Very severe	9 (4.5)	
Waking up too early	None	110 (55.0)	
	Mild	27 (13.5)	
	Moderate	30 (15.0)	
	Severe	25 (12.5)	
	Very severe	8 (4.0)	

SD: standard deviation; BMI: body mass index; BMD: bone mineral density; ISI: Insomnia Severity Index.

A significant positive correlation was found between disease duration and ISI scores (r=0.147, p=0.038). Vitamin D levels showed no significant association with ISI (r=-0.003, p=0.970), FSS (r=-0.026, p=0.714), or HADS depression (r=-0.040, p=0.571) and anxiety scores (r=-0.138, p=0.051). However, serum calcium levels correlated positively with both ISI (r=0.158, p=0.026) and FSS (r=0.166, p=0.019) scores. No significant relationship was observed between BMD or T-scores and sleep, fatigue, or mood. Patients with higher ISI scores also had significantly higher FSS (r=0.342, p<0.001), HADS depression (r=0.297, p<0.001), and HADS anxiety (r=0.323, p<0.001) scores.

There were no statistically significant differences between the ISI ≥15 and ISI <15 groups in terms of femoral neck BMD, L1–L4 BMD, femoral neck T-scores, or L1–L4 T-scores. HADS depression (p=0.011), HADS anxiety (p<0.001), and FSS (p<0.001) scores were significantly higher in the ISI ≥15 group. To aid clinical interpretation, Cohen's d was calculated: fatigue showed a large effect (d=0.831), anxiety a medium-to-large effect (d=0.680), and depression and femoral neck BMD small-to-moderate effects (d=0.383 and 0.444). These results suggest that the differences are not only statistically significant but also clinically meaningful, especially regarding functional and psychological outcomes (Table 2).

**Table 2:** Comparison of clinical and psychological characteristics based on insomnia severity.

	Mean±SD						p-value	Effect size
	ISI<15 (n=159)			ISI≥15 (n=41)				
Age (years)	62.04	±	7.32	62.44	±	6.27	0.700	0.056
Height (cm)	155.87	±	6.55	156.32	±	7.45	0.943	0.067
Weight (kg)	64.63	±	10.86	65.93	±	10.59	0.494	0.120
BMI (kg/m^2^)	26.68	±	4.70	26.98	±	4.03	0.579	0.066
Femoral neck BMD	0.71	±	0.09	0.75	±	0.09	0.087	0.444
L1–L4 BMD	0.74	±	0.08	0.73	±	0.06	0.373	-0.131
L1–L4 T score	-2.86	±	0.55	-2.95	±	0.51	0.178	-0.166
Femoral neck T score	-1.68	±	0.77	-1.44	±	0.78	0.093	0.311
25-OH-D (ng/mL)	30.05	±	13.51	28.65	±	12.51	0.463	-0.105
Calcium	9.63	±	0.47	10.04	±	2.5	0.584	0.342
Disease duration	5.69	±	6.13	6.1	±	6.74	0.636	0.066
VAS	41.76	±	33.65	64.88	±	30.09	***<0.001***	0.694
Coffee (cup/day)	0.89	±	0.96	0.98	±	0.88	0.386	0.095
HADS depression score	6.6	±	4.69	8.34	±	3.92	***0.011***	0.383
HADS anxiety score	7.23	±	4.4	10.24	±	4.54	***<0.001***	0.680
FSS	4.09	±	1.89	5.56	±	1.17	***<0.001***	0.831

Mann-Whitney U test. BMI: body mass index; BMD: bone mineral density; VAS: Visual Analog Scale, HADS: Hospital Anxiety and Depression Scale; FSS: Fatigue Severity Scale; ISI: Insomnia Severity Index; SD: standard deviation. Bold italics indicate statistically significant results at p<0.05.

To assess potential confounders, univariate logistic regression showed significant associations between clinical insomnia (ISI ≥15) and FSS, pain presence, VAS, HADS-D, HADS-A, and femoral neck BMD. The multivariate model included these variables, along with diabetes mellitus and low-energy fracture history based on clinical relevance. Due to multicollinearity between pain presence and intensity, only VAS was retained. In the adjusted model, only fatigue severity (OR 1.702, 95%CI 1.233–2.350, p=0.001) and pain intensity (OR 1.014, 95%CI 1.001–1.027, p=0.030) remained independently associated with insomnia. Femoral neck BMD, which was initially significant, lost statistical significance in the multivariate model (p=0.064) and presented an exaggerated odds ratio with a wide confidence interval, suggesting possible scale-related instability. HADS-D and HADS-A also did not retain significance in the adjusted model (Table 3).

**Table 3:** Univariate and multivariate logistic regression analysis of factors associated with clinical insomnia (Insomnia Severity Index≥15) in postmenopausal women with osteoporosis.

	Β	Univariate model		p-value		Multivariate model			
		OR	95%CI			Β	OR	95%CI	p-value
Age (years)	0.008	1.008	0.960–1.058	0.750					
BMI (kg/m^2^)	0.014	1.015	0.942–1.093	0.705					
VAS	0.022	1.023	1.011–1.035		***0.000***	0.014	1.014	1.001–1.027	***0.030***
Pain (yes)	1.145	3.142	1.162–8.497		***0.024***				
Hypertension	0.001	1.001	0.495–2.024		0.997				
Diabetes mellitus	-1.243	0.288	0.065–1.275		0.101	-1.497	0.224	0.046–1.085	0.063
Gastrointestinal diseases	-0.592	0.553	0.182–1.685		0.553				
CAD	0.234	1.263	0.469–3.399		0.644				
Hypothyroidism	-0.221	0.802	0.308–2.089		0.652				
Coffee (cup/day)	0.099	1.104	0.772–1.579		0.588				
Smoking (yes)	0.426	1.531	0.720–3.253		0.268				
Exercise (yes)	-0.497	0.608	0.199–1.862		0.384				
Low energy fracture (yes)	-0.672	0.511	0.186–1.401		0.192	-0.558	0.572	0.188–1.743	0.326
25-OH-D	-0.008	0.992	0.966–1.019		0.546				
Calcium	0.272	1.312	0.835–2.062		0.239				
Femoral neck BMD	4.087	59.581	1.293–2744.8		***0.036***	4.087	59.575	0.791–4489.4	0.064
L1–L4 BMD	-2.346	0.096	0.001–13.921		0.356				
FSS	0.579	1.785	1.357–2.347		***0.000***	0.532	1.702	1.233–2.350	***0.001***
HADS depression score	0.081	1.084	1.007–1.167		***0.032***	-0.066	0.936	0.829–1.057	0.288
HADS anxiety score	0.143	1.153	1.067–1.247		***0.000***	0.096	1.101	0.987–1.228	0.085

BMD: bone mineral density; HADS: Hospital Anxiety and Depression Scale; FSS: Fatigue Severity Scale; CAD: coronary artery disease; OR: odds ratio; CI: confidence interval; BMI: body mass index; VAS: Visual Analog Scale. Bold italics indicate statistically significant results at p<0.05.

A mediation analysis was performed following the steps of Baron and Kenny. Insomnia significantly predicted fatigue severity (B=0.102, p<0.001) and also significantly predicted depression and anxiety scores (B=0.173 and B=0.229, respectively, p<0.001). When both insomnia and HADS scores were entered into the model predicting FSS, the effect of insomnia was attenuated but remained significant (B=0.076 for depression model, B=0.068 for anxiety model). These findings suggest that both depression and anxiety partially mediate the relationship between insomnia and fatigue in postmenopausal osteoporotic women.

## DISCUSSION

In this study, 20.5% of the 200 postmenopausal women had ISI scores ≥15, indicating significant insomnia symptoms. Among participants, 73.5% experienced difficulty initiating sleep, 57.5% had trouble maintaining sleep, and 45% reported involuntary early awakening. ISI scores were significantly correlated with the severity of fatigue, depression, and anxiety symptoms.

In the study by Yazıcı et al.^12^, 43.8% of 32 postmenopausal osteoporotic women had poor sleep quality, compared to 14.8% in the non-osteoporotic control group. These women also had longer sleep latency and shorter sleep duration. In another study by Şahin Onat et al.^3^, which included both osteoporotic men and women, 42.2% of 154 patients had sleep disturbances. In our study, 20.4% of osteoporotic women had ISI scores ≥15, indicating significant insomnia symptoms. Şahin Onat et al.^3^ also found that poor sleep quality was associated with older age, female gender, being unmarried, unemployment, and illiteracy, while no significant differences were observed regarding smoking, physical activity, or BMI. In contrast, our study did not find significant associations between ISI scores and age, BMI, marital status, occupation, or education level. Although our findings are consistent with previous studies conducted in the Turkish population, it is important to note that geographic region, cultural background, and habitual sleep behaviors may influence perceived sleep quality and insomnia-related outcomes across different populations. For example, in Japan, average sleep duration has been reported to be shorter than in many other countries. In the study by Yamaura et al.^13^, 80% of participants reported sleeping less than 7 h per night, and no significant association was found between insomnia and BMD. In a multiethnic study by Chen et al.^14^, the prevalence of insomnia was lowest among Chinese participants when compared to White, Black, and Hispanic groups. In a Mexican population-based study by Meneses et al.^15^, 73.3% of men and 66.1% of women reported taking regular daytime naps, and longer siesta durations were associated with increased BMD among women. These findings highlight that sleep duration, timing, and perceptions of sleep quality or insomnia can vary widely depending on sociocultural norms and lifestyle. Although our study did not primarily assess total sleep duration or daytime sleep (e.g., napping), the cultural context in which individuals define their sleep as "poor" or "insufficient" may differ across countries. This could partly explain the variability in associations between insomnia and osteoporosis seen in the literature and should be considered when interpreting our findings.

During the conduct of our study, participants were selected solely from individuals presenting to outpatient clinics, and therefore it was inevitable that patients who had not sought medical care due to severe insomnia, fatigue, pain, or mobility limitations may have been overlooked. A recent large-scale longitudinal cohort study conducted by Zhao et al.^16^, involving 293,164 participants, demonstrated that unhealthy sleep behaviors were associated with an increased risk of developing osteoporosis. Similarly, a population-based cross-sectional study conducted in Campinas, Brazil reported that insomnia and short sleep duration (<7 h) were significantly associated with self-reported osteoporosis among older adults^17^. These population-based findings support the relevance of the associations observed in our clinic-based sample and suggest that our results may be generalizable to broader community-dwelling populations.

In our study, insomnia levels were not associated with femoral neck or L1–L4 BMD, contrasting with several studies. A study of 190 postmenopausal Indian women found greater BMD and trabecular bone score loss in those with insomnia (WHIIRS≥9) over 2 years^18^. Fu et al.^6^ also identified a link between shorter sleep duration and lower BMD scores in middle-aged and older women. Similarly, another study showed that postmenopausal women with shorter sleep durations had lower BMD scores^5^. However, some studies align with our findings. Yamaura et al.^13^ found no association between insomnia and BMD in 896 men and 821 women, while Swanson et al.^19^ reported no link between nocturnal sleep duration and BMD in postmenopausal women. These discrepancies may stem from differences in population characteristics and sleep assessment methods.

In our study, it was shown that the levels of depression, anxiety, and fatigue in patients diagnosed with insomnia were correlated with the severity of insomnia. Insomnia is closely related to depressive mood, with early morning awakening being one of the hallmark depressive symptoms^20^. Tal et al.^21^ found significant improvement in depressive symptoms after effective treatment of sleep problems in postmenopausal women. Similarly, Kalmbach et al.^22^ reported that treating insomnia in postmenopausal women led to a reduction in maladaptive thinking and depression levels. Another study also found a strong association between poor sleep quality and depressive symptoms in postmenopausal women^23^. Shim et al.^24^ identified depression and fatigue as predictors of poor sleep quality. In contrast, Yazıcı et al.^12^ found no significant differences in anxiety or depression between osteoporotic and non-osteoporotic women, nor any link between poor sleep and mood, possibly due to their limited sample size.

Our study has both strengths and limitations. One key strength is that it is among the few studies specifically evaluating insomnia in postmenopausal osteoporotic women. Additionally,we utilized the ISI, a validated and widely used scale, and to our knowledge, no previous study has applied it in this specific population, underscoring the novelty of our research. By focusing solely on osteoporotic patients, we were able to clearly examine the relationship between sleep disturbances and relevant clinical risk factors. Furthermore, conducting the study across two centers enhances the generalizability of our findings. However, several limitations should be acknowledged. First, only female patients were included, which limits the generalizability of the findings to the male population. Second, the study included only individuals who presented to outpatient clinics, which may have excluded patients with more severe insomnia, pain, or functional limitations who were unable or unwilling to seek medical care. Third, we were unable to evaluate circadian rhythm disturbances, which may play a key role in the pathogenesis of insomnia. Objective assessments such as melatonin level measurement or actigraphy could not be performed due to feasibility constraints. In addition, our sleep assessments were limited to subjective tools and did not include objective measures such as polysomnography or parameters like rapid eye movement (REM) latency or apnea events. Another limitation is the absence of postural assessments. Postural deformities such as kyphosis or scoliosis may cause positional discomfort during sleep and negatively affect sleep quality. However, postural analysis was not conducted in this study. Moreover, all participants were recruited from the Turkish population, and sleep-related perceptions and habits may vary across cultures and geographical regions. Although we discussed our findings in the context of international studies, we were unable to directly evaluate the impact of cultural, societal, or regional differences on sleep perception. Due to the cross-sectional design of our study, causal relationships between insomnia, fatigue, and psychological symptoms cannot be established. To explore potential underlying mechanisms, we performed a mediation analysis to assess whether mood symptoms partially mediated the association between insomnia and fatigue. While our findings suggested partial mediation, the cross-sectional nature of the data limits causal interpretation. Future longitudinal and cross-cultural studies with larger and more diverse samples—including male participants—and the use of objective sleep and activity monitoring tools are warranted to validate and expand upon our results.

## CONCLUSION

In conclusion, insomnia and poor sleep quality are significantly related to an increased risk of falls and, consequently, a higher likelihood of fractures in osteoporotic patients. Therefore, early detection of sleep disturbances and appropriate interventions in osteoporotic individuals are crucial to prevent future complications. Further studies with larger sample sizes and more comprehensive evaluation methods are needed to support and expand upon these findings.

## Data Availability

The datasets generated and/or analyzed during the current study are available from the corresponding author upon reasonable request.
